# Design of Spiking Central Pattern Generators for Multiple Locomotion Gaits in Hexapod Robots by Christiansen Grammar Evolution

**DOI:** 10.3389/fnbot.2016.00006

**Published:** 2016-07-28

**Authors:** Andres Espinal, Horacio Rostro-Gonzalez, Martin Carpio, Erick I. Guerra-Hernandez, Manuel Ornelas-Rodriguez, Marco Sotelo-Figueroa

**Affiliations:** ^1^Leon Institute of Technology, Leon, Mexico; ^2^Department of Electronics, DICIS-University of Guanajuato, Salamanca, Mexico; ^3^Department of Organizational Studies, División de Ciencias Economico-Administrativas-University of Guanajuato, Guanajuato, Mexico

**Keywords:** central pattern generator, spiking neural network, Christiansen grammar evolution, evolution strategy, SPIKE-distance, legged robot locomotion, FPGA

## Abstract

This paper presents a method to design Spiking Central Pattern Generators (SCPGs) to achieve locomotion at different frequencies on legged robots. It is validated through embedding its designs into a Field-Programmable Gate Array (FPGA) and implemented on a real hexapod robot. The SCPGs are automatically designed by means of a Christiansen Grammar Evolution (CGE)-based methodology. The CGE performs a solution for the configuration (synaptic weights and connections) for each neuron in the SCPG. This is carried out through the indirect representation of candidate solutions that evolve to replicate a specific spike train according to a locomotion pattern (gait) by measuring the similarity between the spike trains and the SPIKE distance to lead the search to a correct configuration. By using this evolutionary approach, several SCPG design specifications can be explicitly added into the SPIKE distance-based fitness function, such as looking for Spiking Neural Networks (SNNs) with minimal connectivity or a Central Pattern Generator (CPG) able to generate different locomotion gaits only by changing the initial input stimuli. The SCPG designs have been successfully implemented on a Spartan 6 FPGA board and a real time validation on a 12 Degrees Of Freedom (DOFs) hexapod robot is presented.

## 1 Introduction

Since the early twentieth century, studies have been carried out to explain how the rhythms of locomotor movements in living beings are created (Brown, 1911). It was proposed that the action of walking is carried out by neural mechanisms, in which neurons are inhibiting each other to achieve the control of muscles achieving a rhythmic movement (Brown, 1914). Nowadays there is evidence supporting this idea, behavior-based studies of living beings have demonstrated that neural mechanisms, known either as neural oscillators or CPGs, contribute to locomotion. Although it has been experimentally demostrated that CPGs can endogenously produce rhythmic motor outputs, they do not work isolatedly; CPGs also depend on the information interaction with other parts of the central nervous system (Arena, 2000). Moreover, afferent sensory inputs are used to shape the final motor output (MacKay-Lyons, 2002). CPGs produce other rhythmic behaviors without conscious effort besides locomotion, including respiration, heart beat, swallowing, etc. (Patel, 2009).

Studies about CPGs have produced several mathematical models of such mechanisms, which have been used in both theoretical and practical fields for different purposes. Recently, in robotics, there has been an increasing interest in the design and implementation of biologically inspired CPG-based locomotion systems (Russell et al., 2007; Crespi and Ijspeert, 2008; Wyffels and Schrauwen, 2009; Barron-Zambrano and Torres-Huitzil, 2012; Chen et al., 2012; Hong et al., 2014; Nassour et al., 2014; Park et al., 2014; Rostro-Gonzalez et al., 2015) with rhythmic motions instead of non-biologically plausible methods such as those based on finite-state machines, sine-generators, pre-recorded reference trajectories (Vukobratović and Borovac, 2004) or heuristic control laws (Pratt et al., 2001). There are features of CPGs, which make them suitable as locomotion systems in robotic controllers (Yu et al., 2014); they ensure a uniform and steady rhythm over course of locomotion, they possess stability that makes them robust against disturbances, they can be modified by the sensory feedback signals by means of their behavioral adaptability and one of them can generate different motor behaviors by switching between behaviors arising from changes in parameters.

**Figure 1:**
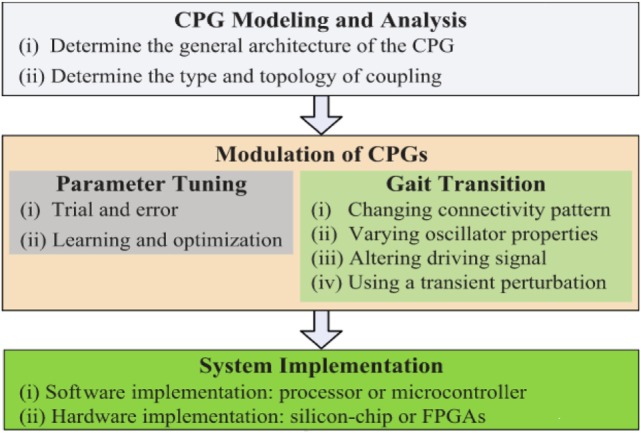
**Workflow diagram of CPG design taken from Yu’s work (Yu et al., 2014)**.

Even though several implementations of CPG-based locomotion systems for robots have been reported in the state of the art [see Ijspeert (2008), Wu et al. (2009), and Yu et al. (2014) for detailed reviews on CPG research], there is a lack of a well-established design methods for CPG systems (Ijspeert, 2008); however, a generic framework for designing CPGs is proposed in Yu et al. (2014) based on three main aspects on which most CPG studies have focused (see Figure 1):

1. *CPG Modeling and Analysis*: This task deals with choosing the type of neuron or oscillator, the kind of coupling (unidirectional or bidirectional connection), and the structure of the connections.
2. *Modulation of CPGs*: In engineering, two components of CPG modulation are used: the parameter tuning and the gait transition. The former is usually achieved by using trial-and-error optimization methods (deterministic and stochastic). The latter component deals with methods for handling several gaits generated by a CPG. Some common approaches implemented to handle the generation of several gaits for CPGs are: changing the pattern of connectivity, varying the properties of the oscillators (reconfiguration), altering the driving signal to the CPG, and using a transient disturbance (environmental adaptability).
3. *System Implementation*: CPG-based locomotion systems can be programed in software and they can run on a micro-controller or in a reconfigurable hardware, such as field-programmable gate array (FPGAs) or neuromorphic systems.

Lately, a CPG development method for hexapod robot locomotion systems, which defines the CPG modeling (network topology) and the CPG parameter tuning by means of a reverse-engineering approach to estimate the parameters of a neural network has been developed (Rostro-Gonzalez et al., 2012); in that study, gait transition is made by changing connectivity patterns because a CPG is created for each gait and finally, they are tested on hardware (Rostro-Gonzalez et al., 2015). In this study, we propose a method based on the CPG design published in the aforementioned study. Our design methodology is based on Christiansen Grammar Evolution (CGE) (Ortega et al., 2007), a kind of optimization algorithm with indirect representation of solutions, which can be used for the development of the Evolutionary Artificial Neural Networks (Yao, 1999; Ding et al., 2013); by using CGE the method dispenses with predefined topology and avoids an explicit training process, which is a difficult task because certain parameters need to be estimated to replicate locomotor patterns (Ijspeert, 2008; Buschmann et al., 2015). CGE defines the presynaptic connections and the weights of a spiking neuron (see Section 2.2.1); once a spiking neuron is connected, its capability of replicating a specific signal is valued by SPIKE-distance (Kreuz et al., 2013). The methodology integrates all the individual design to define a whole CPG. We are capable of generating compact CPG topologies and to create a CPG which generate different gaits.

In legged robots, locomotion can be performed by CPGs, which are mainly described by oscillators or artificial neurons with a lack of biological plausibility, e.g., connectionist models, vector maps and systems of coupled oscillators (Ijspeert, 2008). Recently, there are few efforts to implement SCPGs as locomotion controllers in robotics. SPCGs are built as Spiking Neural Networks (SNNs), the third generation of Artificial Neural Networks (ANNs) (Maass, 1997); these are formed by spiking neurons, whose models are biologically plausible and can process spatio-temporal information naturally as required for rhythmic movements. Some SCPGs have been designed and implemented for locomotion on biped and hexapod robots; in Lewis et al. (2005), an architecture of spiking neurons to generate walking gaits for a biped robot was proposed. Later, Russell et al. (2010) proposed to implement a Genetic Algorithm (GA) to reconfigure weights and network topology of Lewis’ network online for changing the locomotion on the same kind of robot. More recently in Rostro-Gonzalez et al. (2015), SNNs are configured as CPGs based on an analysis of six-legged insects’ gaits for hexapod robot locomotion. In this work, SCPG-based locomotion systems are designed to imitate different gaits observed in hexapod insects, which are implemented in an FPGA Spartan 6 board and tested on hexapod robots.

There are reasons that encourage the study, design, and implementation of SCPGs, e.g. the advances on interfacing prosthetic robotic devices to amputated humans and spinal injury patients; and because SCPG are made of SNNs, which receive and process the same kind of information as the CPGs on their biological counterpart, they are a reliable and viable option (Russell et al., 2007). The structure of the paper is as follows. In Section 2, the proposed methodology is presented. In Section 3, we present the experimental configuration and the numerical results. Finally, in Section 4, the conclusions and highlights of this study are given.

## 2 Materials and Methods

In this section, we introduce the design methodology of SCPGs and all the required methods for their development. The proposal is an off-line methodology, it is, the algorithm for SCPG design was implemented using JAVA as its programing language then we developed hardware architecture based on VHDL (VHSIC Hardware Description Language) for FPGA targeting and real time simulation on a hexapod robot. The design methodology is a system of inputs, design process and output. The inputs are sets of rhythmic patterns for locomotion of hexapods (see Section 2.1), each of them explicitly defining the periodic signal for each spiking neuron into the SCPG. The design process is based on the divide-and-conquer approach, where a problem is divided into subproblems that are independently solved to be combined into a solution of a whole (Puntambekar, 2008). Here, the problem is to design an SNN (see Section 2.2) that endogenously replicates the input rhythmic patterns. Since the SCPG is built as an SNN, it needs to replicate specific rhythmic patterns to contribute to locomotion, and thus it is very important that each neuron may be able to replicate its expected signal periodically through the connections with other neurons (or even with itself). The design of an SNN for producing rhythmic patterns is achieved by dividing the general design of the SNN into individual ones for each spiking neuron. The spiking neuron design is created by using its initial rhythmic signal’s state and the signals from other neurons through CGE (see Section 2.3), which codifies information into solutions such as connections and weights; then, they are perturbed until the spiking neuron replicates the expected signal. Several design specifications can be integrated into the fitness function of the CGE. The output is an SCPG obtained by integrating all the individual neuron designs. Finally, the designed SCPGs are implemented on a Spartan 6 FPGA board and tested on an hexapod robot (see Section 2.4).

### 2.1 Hexapod Locomotion Gaits

**Figure 2:**
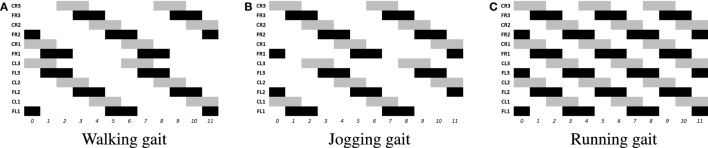
**Rhythmic patterns for hexapod locomotion**. The black bars correspond to the gait patterns reported in Grabowska et al. (2012), used for moving the femurs, while the gray bars are added in Rostro-Gonzalez et al. (2015) as additional information for moving the coxas and thus achieving the full locomotion in hexapod individuals. **(A)** Walking gait. **(B)** Jogging gait. **(C)** Running gait.

The locomotion of hexapods is achieved by designing SCPGs that are periodically able to replicate rhythmic signals, which contribute to imitate gaits observed in real hexapod insects. To drive the SCPG designs, samples from the rhythmic signals are required; here, three different gait patterns are used [proposed in Rostro-Gonzalez et al. (2015)]. These biologically based patterns are the result of a study about the inter-leg coordination of stick insect (*carausius morosus*) on free walks (Grabowska et al., 2012); in this study, two tetrapod gaits and one tripod gait were reported, labeled in Rostro’s work as walking gait (Figure 2A), jogging gait (Figure 2B), and running gait (Figure 2C), respectively. In Figure 2, the three locomotion patterns are presented, where the *x* and *y* axes show the scale of time (as reference) and neuron labels (according with Figure 4), respectively. In such figure, each bar represents the neural activity that stimulates a servomotor in the robot, where Coxa-Left (CL) and Coxa-Right (CR) from 1 to 3 correspond to the servomotors for the coxa (hip joint) of the robot, it is, the articulation in charge of the movement from back to front and viceversa. On the other hand, Femur-Left (FL) and Femur-Right (FR) from 1 to 3 correspond to the servomotors for the femur of the robot, it is, the articulation in charge of the movement from down to up and viceversa. The coordinate movements of these two articulations perform the expected locomotion gaits such as those shown in Figure 2.

### 2.2 Spiking Neural Network

Herein, the CPGs are built as SNNs, similar to other ANNs, and can be defined around three aspects: neuron model, synaptic connections, and message types (Judd, 1990). For this study, we used the simplest form of the integrate-and-fire spiking neuron model, which is based on the discrete-time representation of the membrane potential of the neuron and called BMS neuron model (Soula et al., 2006) (see Section 2.2.1), the synaptic connections are both excitatory (positive values) and inhibitory (negative values) and they are defined by the CGE (see Section 2.3) for each neuron, and finally the message types are spike times.

#### 2.2.1 BMS Neuron Model

The BMS spiking neuron model (Soula et al., 2006) is a discrete-time version of the best-known and widely used generalized Integrate-and-Fire model (gIF) (Gerstner and Kistler, 2002). In the BMS model, the membrane potential *V_i_* and the firing state *Z_i_* of the *i*th neuron at time *k* are given by equations (1) and (2), respectively.

(1) $$V_{i}\left[k\right]=\gamma V_{i}\left[k-1\right]\left(1-Z_{i}\left[k-1\right]\right)+\sum_{j=1}^{N}\;W_{\mathrm{ij}}Z_{j}\left[k-1\right]+I_{i}^{\mathrm{ext}}$$

(2) $$Z_{i}\left[k\right]=\left\{\begin{array}{cc} 1\; & \text{if }V_{i}\left[k\right]\geq \theta \\ 0\; & \text{otherwise} \end{array}\right.$$

where *γ* ∈ [0, 1] defines the leak rate. *N* is the number of neurons in the neural network. *W* is the matrix of synaptic weights. Finally *I*^(^*^ext^*^)^ represents an external stimulus, but here *I*^(^*^ext^*^)^ = 0 because SCPGs endogenously generate the rhythmic patterns. When *V_i_*[*k*] reaches a given threshold *θ*, then a spike occurs in *Z_i_*[*k*] equation (2), and the neuron *i* is reset by the term (1 − *Z_i_*[*k*]) in equation (1).

### 2.3 Christiansen Grammar Evolution

The Christiansen Grammar Evolution (CGE) (Ortega et al., 2007), like the Grammatical Evolution (GE) (Ryan et al., 1998), is a grammar-based form of Genetic Programing (GP) (Koza, 1992). CGE extends the capabilities of GE in the sense that it can generate both syntactically and semantically correct programs. This is achieved by replacing the Context-Free Grammars with Christiansen Grammars (CG) (Christiansen, 1985).

The CGE can be explained from the basis of GE as follows: the genotypic representation of individuals is lineal, formed by strings of numeric values. These are changed from their genotype representation to their functional phenotypic representation through a mapping process (Dempsey et al., 2009) (also known as indirect representation), which uses a grammar to derive the phenotypic representation of an individual. The search process is made using a search engine (usually a metaheuristic algorithm), which modifies the genotype of the individual with only the knowledge of its fitness value. Since CGE has been inspired from GE, their work flows are similar with the exception of the kind of input grammars and some extra steps on the mapping process (see Sections 2.3.1 and 2.3.2, respectively).

Recently, based on the fact that several algorithms have been used as GE search engines [i.e., Genetic Algorithm (Ryan et al., 1998), Differential Evolution (O’Neill and Brabazon, 2006a), Particle Swarm Optimization (O’Neill and Brabazon, 2006b)], a generic methodology for implementing GE was suggested, which points out their input conditions (problem instance, BNF grammar, and search engine) and process cycle (output or individual’s phenotypic representation given by the mapping process and its evaluation by using a fitness function) (Sotelo-Figueroa et al., 2014). From the relationship between GE and CGE, this proposal can be easily adapted for CGE by changing the kind of input grammar and the mapping process.

#### 2.3.1 Christiansen Grammars

The Christiansen Grammars (CGs) (Christiansen, 1985) are adaptable grammars, which can be modified on the fly while they are being used. According to Shutt’s work (Shutt, 1993), CGs are very similar to Extended Attribute Grammars (Watt and Madsen, 1983); CGs are defined as a 5-tuple as follows: CG = {Σ*_T_*, Σ*_N_*, *S*, *P*, *K*}, where Σ*_T_* is the set of terminals, Σ*_N_* is the set of non-terminals, *S* ∈ Σ*_N_* is the axiom of the grammar, *P* is the productions set, and *K* is the global information of the grammar.

The key features of the CGs syntax (Ortega et al., 2007) are:

- The non-terminal symbols are written between angled brackets and they are followed by a parenthesized list of their attributes. The value of any attribute can be computed while the grammar is being used. The first attribute of each non-terminal is a CG, which contains the applicable rules to the corresponding symbol.
- The attributes can be either inherited (↓) or synthesized (↑).
- In the production rules, the semantic actions follow their corresponding production rule in brackets, where {} stands for non-semantic action. These actions are usually written in pseudocode.

**Figure 3:**
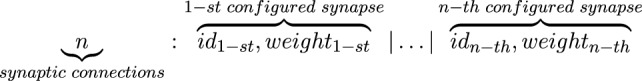
**Structure of the derived word for presynaptic connections of a neuron**.

For this study, we propose a CG in order to derive words that represent the presynaptic connectivity and configuration of a single spiking neuron. The neuron can be connected with itself and other neurons. However, a neuron can only allow one connection per neuron in the network; this means that a neuron must have at least one connection and the maximum number of connections is the number of neurons in the network. Figure 3 shows the structure of a derived word that represents the *n* configured presynaptic connections of a postsynaptic neuron.

Next, the CG for defining presynaptic connections of a neuron is introduced.

The set of terminals Σ*_T_* = {1, 2, … , 8, 9, … , *N* − 1, *N*, +, −, :, |} includes all the characters accepted for a valid word; numbers from 1 to *N* (number of servomotors in the hexapod robot) are used to define the number of connections or the index of a presynaptic neuron (the labeled servomotors are associated to an index for simplicity of the word), numbers from 1 to 9 are used to define synaptic weights, symbols + and − are used for excitatory and inhibitory weights respectively and symbols : and | are auxiliary for parsing the word.

The set of non-terminals Σ*_N_* = {

***<neuronSynapses****>*** (***↓****g_i_*)**,
***<connections****>*** (***↓****g_i_*, ***↑*** *g_o_*)**,
***<neuronIdList****>*** (***↓****g_i_*, ***↑*** *n*)**,
***<synapses****>*** (***↓****g_i_*)**,
***<synapse****>*** (***↓****g_i_*, ***↑*** *g_o_*)**,
***<weight****>*** (***↓****g_i_*)**,
***<sign****>*** (***↓****g_i_*)**,
***<digit****>*** (***↓****g_i_*)**
}

The axiom of the grammar *S* = ***<neuronSynapses****>*** (***↓****g_i_*)**.

The production set *P* = {

***<neuronSynapses****>*** (***↓****g_i_*) ⊨ ***<****connections****>*** (***↓****g_i_*, ***↑*** *g_o_*): ***<****synapses****>*** (***↓****g_o_*)** {},
***<connections****>*** (***↓****g_i_*, ***↑*** *g_o_*) ⊨ ***<****neronIdList****>*** (***↓****g_i_*, ***↑*** *n*)** {
↑ g_o_ = ↓ g_i_ ∪ <synapses> (↓ g_i_) ⊨ <synapse> (↓ g_i_, $↑{\text{ g}}_{{\text{o}}_{1}}$) |…| <synapse> $\left(↓g_{o_{↑n-1}}↑\;g_{o_{↑n}}\right)$ {}
},
***<neuronIdList****>*** (***↓****g_i_*, ***↑***1)** ⊨ 1{},
…
***<neuronIdList****>*** (***↓****g_i_*, ***↑*** *N*)** ⊨ *N*{},
***<synapse****>*** (***↓****g_i_*, ***↑*** *g_o_*) ⊨ ***<****neuronIdList****>*** (***↓****g_i_*, ***↑*** *n*), ***<****weight****>*** (***↓****g_i_*)** {
↑ g_o_ = ↓ g_i_ − { <neuronIdList> (↓ g_i_, ↑ n) ⊨ ↑n{}}},
***<weight****>*** (***↓****g_i_*) ⊨ ***<****sign****>*** (***↓****g_i_*)***<****digit****>*** (***↓****g_i_*)** {},
***<sign****>*** (***↓****g_i_*)** ⊨ +{},
***<sign****>*** (***↓****g_i_*)** ⊨ −{},
***<digit****>*** (***↓****g_i_*)** ⊨ 1{},
…
***<digit****>*** (***↓****g_i_*)** ⊨ 9{}
}

There is no global information for this CG, thus *K* = ∅.

#### 2.3.2 CGE Mapping Process

The CGE Mapping Process is a deterministic method that transforms individuals from their genotype form into their phenotype form; this allows individuals to be evaluated in the problem context through a fitness function. The genotype-to-phenotype mapping process for the CGE is carried out as follows (Ortega et al., 2007):

1. Choose the leftmost non-terminal symbol in the sentential form being processed.
  1.1. Evaluate the attributes.
  1.2. Select the applicable rules from the first attribute in each non-terminal.
2. Number the *n* right-hand sides of all the rules for this non-terminal symbol (from 0 to *n* − 1), where the rules are in an arbitrary order, which should be maintained during the whole process.
3. If *n* > 1, select the right-hand side of the rule whose number equals codon mod (number of right-hand sides for this non-terminal). Else if *n* = 1, then the unique rule is selected, and the codon is not consumed.
4. Derive the next word by replacing the non-terminal with the selected right-hand side.

In Appendix A, there is an example of a derivation tree generated by the CGE mapping process and the proposed CG.

#### 2.3.3 Search Engine: (1 + 1) – Evolution Strategy

The Evolution Strategies (ESs) (Rechenberg, 1973; Schwefel, 1977) are optimization algorithms based on the concept of the evolution of evolution, because biological processes have been optimized by evolution, and evolution is a biological process in itself (Engelbrecht, 2007). This family of algorithms is part of Evolutionary Algorithms (EAs).

In the literature, several variants of ES algorithms have been proposed. They can be generally described by the following notation (*μ*/*ρ*$\overset{+}{,}$*λ*) – ES. The *μ* is the size of the parent population, *ρ* is the number of parents in the crossover, λ is the size of the offspring population and ($\overset{+}{,}$)-selection operators indicate from which population (s) is (are) individuals selected for the next generation of parents; the plus-selection (+) takes into account both populations, while the comma-selection (,) only takes into account the offspring population.

In ES, the candidate solutions of a *d*-dimensional problem are formed by the object parameter vector *y* and the endogenous strategy parameters. The type of components of *y* depend on the problem to solve (ℝ, ℕ, 𝔹 or more complex structures are allowed) (Beyer, 2013), and the number and type of strategy parameters can vary according to the design of the candidate solutions; these strategy parameters are used to self adapt the ES and they are not involved on the fitness calculation of individuals.

For this work (1 + 1) – ES is used; this ES has one parent and only one offspring is generated. It uses the plus-selection operator, whereby the best individual from the parent and its offspring is selected to form the parent population in the next generation. The solutions are formed using the vector *y* and only one strategy parameter *σ*. Algorithm 1 shows the implemented (1 + 1) – ES [based on the theory of ES algorithms (Engelbrecht, 2007; Beyer, 2013)].

**Algorithm 1:** **(1+1) – ES**.

1: initialize ( *y*, *σ*)
2: *F_y_* : = *F*( *y*)
3: **repeat**
4: $\tilde{\sigma }:=\sigma {\text{e}}^{\tau }$**^N(0,1)^**
5: $\tilde{y}:=y+\tilde{\sigma }$(**N(0,1)**_1_, … , **N(0,1)**_*d*_)^*T*^
6: $F_{\tilde{y}}:=F\left(\tilde{y}\right)$
7: **if** $F_{\tilde{y}}<F_{y}$ **then**
8: $y:=\tilde{y}$
9: $\sigma :=\tilde{\sigma }$
10: **end if**
11: **until** stop criterion

In Algorithm 1, line 1, the vector *y* is randomly initialized in the search range for each *y* component (which depends on the problem to solve) and the strategy parameter is set to be *σ* ~ 3.0 (Dortmund, 1995). In line 2, *F*(•) implies the evaluation of an object parameter vector. In line 4, *τ* is a learning parameter used to update *σ*, usually calculated from the dimensionality of the problem $\tau =\frac{1}{\sqrt{d}}$ (Engelbrecht, 2007). Finally, *N*(0, 1) are random numbers normally distributed with mean equals to 0 and a SD of 1 (Talbi, 2009).

#### 2.3.4 SPIKE-Distance-Based Fitness Functions

Since there is no explicit model for fitness computation in the search space of connectivity configuration, we have to explore how to build up one. We have found that an important criteria to achieve such a goal is to explore the aforementioned search space to make a specific neuron replicated an input rhythmic signal. In functional approximation, an alternate and explicit mathematical expression is constructed for the objective function (Jin, 2005), which in this case is unknown.

For this study, two fitness functions were designed as functional approximations to improve in some aspect the topological design of SCPGs; the fitness functions are based on the Bivariate SPIKE-distance *D_S_*(•,•) (Kreuz et al., 2013) (see Appendix B). In general, both fitness functions have the same purpose, to drive the search by measuring the similarity between a target spike train and a generated spike train. But each fitness function takes into account extra criteria, such as structural aspects or number of rhythmic patterns to be replicated. Next, the designed fitness functions are shown, in equation (3), *s_t_* is the target spike train, and *s_g_* is the spike train generated after the simulation of a presynaptic connection design of a neuron. In equation (4), $\left\{s_{t_{i}}\right\}$ is a set of target spike trains, and $\left\{s_{g_{i}}\right\}$ is a set of the spike train generated after the simulation of a presynaptic connection design of a neuron where $i=1,\ldots ,|\left\{s_{t_{i}}\right\}|$ and $\left|\left\{s_{t_{i}}\right\}|=|\left\{s_{g_{i}}\right\}\right|$.

The function given by equation (3) is defined with the intention of achieving compact topologies; it takes into account structural information about the normalized number of connections ($\frac{\mathrm{num}\text{\_}\mathrm{conn}}{N}$, *num*_*conn* is the number of presynaptic neurons connected to the current neuron and *N* is the total number of neurons into the SCPG) required to replicate the input information plus the similarity between spike trains to assign a fitness value to an individual.

(3) $$f_{1}\left(s_{t},s_{g}\right)=D_{S}\left(s_{t},s_{g}\right)+\frac{\mathrm{num}\text{\_}\mathrm{conn}}{N}$$

The function given by equation (4) is defined with the intention of making SCPGs capable of generating several rhythmic patterns (as mentioned in Section 1); it takes into account the accumulation of similarities between spike trains of sets of target spike trains and generated spike trains to assign a fitness value to an individual.

(4) $$f_{2}\left(\left\{s_{t_{i}}\right\},\left\{s_{g_{i}}\right\}\right)=\sum_{i=1}^{\left|\left\{s_{t_{i}}\right\}\right|}D_{S}\left(s_{t_{i}},s_{g_{i}}\right)$$

### 2.4 Hardware

**Figure 4:**
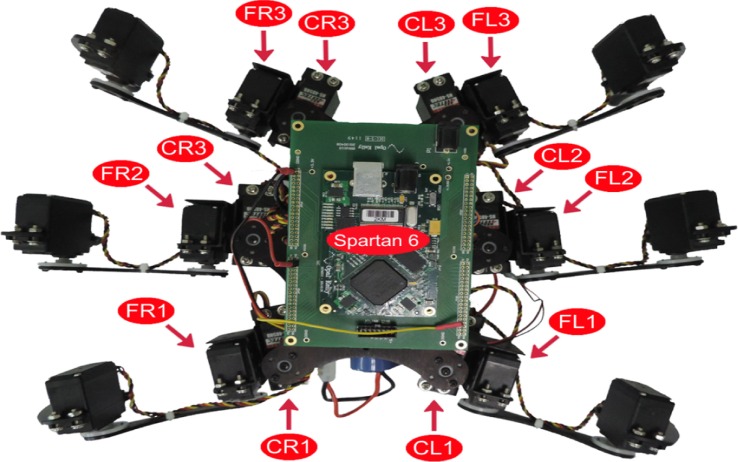
**Hexapod robot with FPGA Spartan 6**.

SCPGs have been implemented in hardware (on an FPGA Spartan 6 board) and successfully validated on a real hexapod robot. The FPGA-based implementation is a fully reconfigurable hardware architecture, which runs the different SCPGs in real time. The FPGA controls all the servomotors (neurons) in the hexapod robot in order to perform the gaits shown in Figure 2. The hexapod robot configuration implemented is shown in Figure 4 (Rostro-Gonzalez et al., 2015); each leg is controlled by two servomotors, i.e., the motors for coxa and femur. Thus, the hardware configuration requires twelve neurons to handle all the servomotors on the implementation.

## 3 Results

For this study, two experiments were carried out, one for each fitness function, to design SCPGs. Next, the parameters for all the experiments are reported.

The configuration parameters are the same for all experiments, unless a particular case is explicitly specified. The configuration was as follows:

- *BMS model*: a normalized BMS neuron was used, and thus the threshold *θ*, in equation (2), was set at a value of 1. The leak rate *γ*, in equation (1), was assigned to 0.5 for ease of implementation on hardware.
- (1 + 1) – ES:
  - The dimension of the search space was *d* = 75.
  - The range of each component of the object parametor vector, *y_j_* ∈ [0, 255] where *j* = 1, …, *d*. In the mapping process, each component value is rounded to the closest integer.
  - The function calls were the stop criteria of the algorithm; the number of function calls for designing each neuron varies according to the fitness function used. In the experiments of the first fitness function, given by equation (3), 50 function calls were used. For the experiment of the second fitness function, given by equation (4), 500 function calls were used.

To validate the design of the SCPGs, numerical tests based on two different fitness functions were carried out, and the results are now presented.

In the first experiment, we considered equation (3) as the fitness function. In this case, we generated an SCPG for each gait; thus the gait transition must be done by changing the connectivity pattern. For this experiment, the expected fitness value on every design is equal to 1; only one presynaptic neuron is expected to stimulate the postsynaptic neuron to reproduce its input signal. The results are given in the following order: walk, jog, and run gaits, respectively. The generated words for presynaptic connectivity and the final topologies resulting from the integration of individuals designs for each gait are reported in Tables 1–3. Finally, the weight matrices for each gait are given in equations (15–17) (see Appendix C). As can be observed, the SCPGs were successfully designed by using this fitness function. The number of synaptic is *N* = 12 (the minimum expected), due to that fact that there is a restriction on the number of these during the computation.

**Table 1:** **Configuration of designed SCPG for walking gait by using equation (3)**.

Neuron (ID)	Presynaptic connectivity	Topology
FL1 (1)	1:2, +4	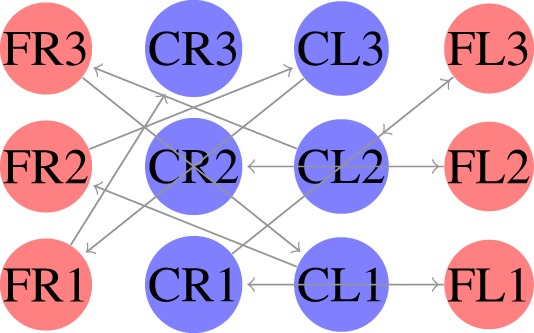
CL1 (2)	1:11, +9	
FL2 (3)	1:4, +3	
CL2 (4)	1:5, +1	
FL3 (5)	1:8, +7	
CL3 (6)	1:9, +6	
FR1 (7)	1:6, +1	
CR1 (8)	1:1, +7	
FR2 (9)	1:2, +6	
CR2 (10)	1:3, +3	
FR3 (11)	1:4, +6	
CR3 (12)	1:7, +5	

**Table 2:** **Configuration of designed SCPG for jogging gait by using equation (3)**.

Neuron (ID)	Presynaptic connectivity	Topology
FL1 (1)	1:2, +5	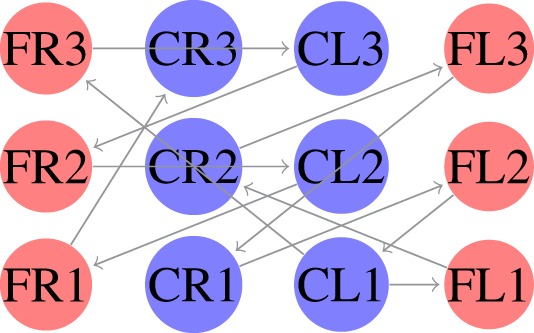
CL1 (2)	1:3, +5	
FL2 (3)	1:8, +1	
CL2 (4)	1: 9, +7	
FL3 (5)	1:10, +9	
CL3 (6)	1:11, +8	
FR1 (7)	1:4, +2	
CR1 (8)	1:5, +5	
FR2 (9)	1:6, +9	
CR2 (10)	1:1, +8	
FR3 (11)	1:2, +2	
CR3 (12)	1:7, +4	

**Table 3:** **Configuration of designed SCPG for running gait by using equation (3)**.

Neuron (ID)	Presynaptic connectivity	Topology
FL1 (1)	1:6, +1	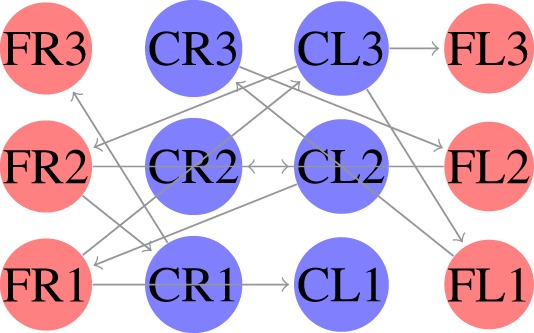
CL1 (2)	1:7, +9	
FL2 (3)	1:12, +7	
CL2 (4)	1:9, +4	
FL3 (5)	1:6, +5	
CL3 (6)	1:7, +6	
FR1 (7)	1:4, +4	
CR1 (8)	1:9, +5	
FR2 (9)	1:6, +1	
CR2 (10)	1:3, +5	
FR3 (11)	1:8, +9	
CR3 (12)	1:1, +9	

In the second experiment, we considered equation (4) as the fitness function. Here, we generated a single SCPG for the three gaits. Thus, the gait transition can be performed by altering the driving signal (switching the state of the SNN for the initial state of the desired gait). For this experiment, the expected fitness value for every design is 0. The generated words for presynaptic connectivity and the final topologies generated by the integration of individuals designs are reported in Table 4. Finally, the weight matrix is given in equation (18) (see Appendix C). As can be observed, the SCPG was successfully designed by using this fitness function. In terms of hardware design, this method is highly suitable to be provided of sensory information as the driving signal.

**Table 4:** **Configuration of designed SCPG for all gaits by using equation (4)**.

Neuron (ID)	Presynaptic connectivity	Topology
FL1 (1)	1:2, +8	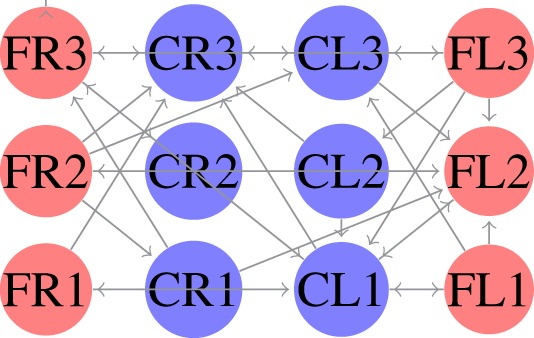
CL1 (2)	7:5, −2|7, +1|8, +2|1, −7|11, +2|3, +4|4, +2	
FL2 (3)	7:6, +1|8, −2|5, +4|9, +4|2, −5|1, −9|4, +8	
CL2 (4)	1:5, +8	
FL3 (5)	1:6, +5	
CL3 (6)	5:9, +3|5, −9|11, +3|12, −1|1, +6	
FR1 (7)	1:8, +5	
CR1 (8)	1:9, +4	
FR2 (9)	1:10, +8	
CR2 (10)	1:11, +9	
FR3 (11)	5:8, −2|2, −4|12, +9|11, +3|6, −3	
CR3 (12)	7:5, +8|2, +1|9, −4|6, −1|4, −3|7, +5|11, −4	

**Figure 5:**
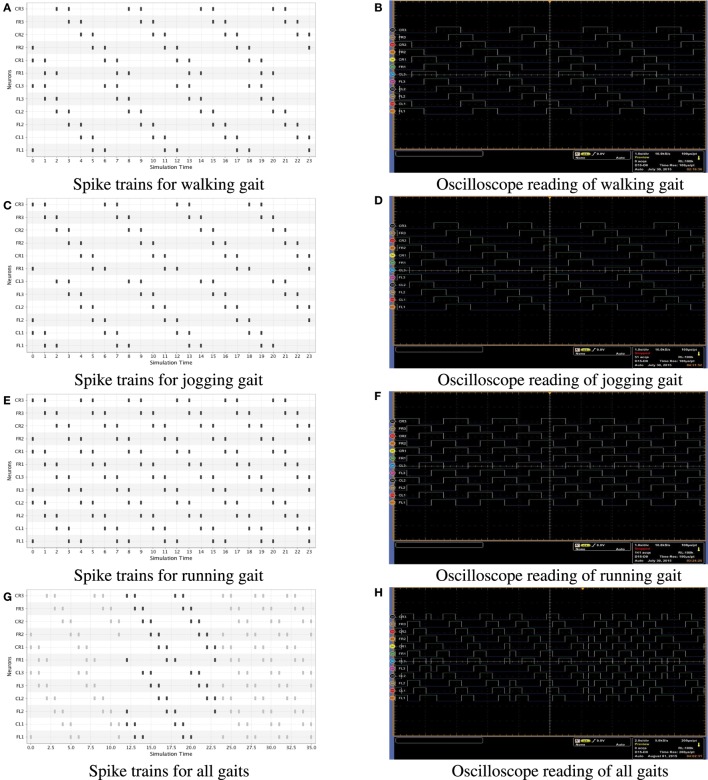
**Spike trains generated for all gaits; Figures (A–F) were obtained by equation (3) and Figures (G,H) were obtained by equation (4)**. In the left side we present numerical simulations in software and on the right side oscilloscope signals in a real time simulation of the hexapod robot. The oscilloscope signals are directly taken from the FPGA. **(A)** Spike trains for walking gait. **(B)** Oscilloscope reading of walking gait. **(C)** Spike trains for jogging gait. **(D)** Oscilloscope reading of jogging gait. **(E)** Spike trains for running gait. **(F)** Oscilloscope reading of running gait. **(G)** Spike trains for all gaits. **(H)** Oscilloscope reading of all gaits.

Finally, in Figure 5, we also present the results of a real-time simulation on the hexapod robot. In this case, we show both the simulation in software (left side) and the oscilloscope signals (right side) generated during the performance of a locomotion pattern (walking, jogging, and running) in the robot. In order to get the real time signals, we used an MSO5204B Mixed Signal Oscilloscope, which has 16 digital channels. We only used 12 of the 18 available servomotors in the robot out of the 16 digital channels, which was enough to perform the measures.

## 4 Conclusion

In this study, an automatic design methodology involving CPGs built as SNNs for hexapod locomotion has been presented. The proposal follows the divide-and-conquer approach to design the SNNs for given input rhythmic signals. Instead of designing the SNN as a whole, the methodology integrates the individual presynaptic configuration (connectivities and weights) of each locomotor neuron to create the final SNNs. The individual presynaptic configuration is carried out by CGE, which modifies the solutions over the search space of connections and weights. The CGE allows to dispense with a predefined architecture to avoid the explicit learning process for a neuron when replicating a specific rhythmic signal. The aforementioned advantage is possible because the solution indirectly represents the number of presynaptic connections, the indexes of these connections and their respective weights. The quality of the solutions is given by fitness functions that are mainly based on SPIKE-distance.

The proposed methodology has been successfully validated by generating SNNs to replicate rhythmic signals. The two fitness functions used in this work allow us to implement the hexapod gaits transitions in two ways: by changing the pattern connectivity when each gait is produced by one designed SNN and by altering the driving signal when an SNN can produce several rhythmic signals. The designed SCPGs were validated in both computer simulations and hardware implementation running on an FPGA to control an hexapod robot and, all of them showed the desired behaviors. Moreover, this study has reached the results reported in Rostro-Gonzalez et al. (2015) by generating one SCPG for each gait and their implementations, and it has managed to generate SCPGs capable of producing several rhythmic signals taking as basis the proposal of Rostro et al.; this was possible due to the characteristics of the EAs and the SPIKE-distance based fitness function definitions.

Our proposal was used for hexapod gaits and their implementation over hexapod robots; however it can be tested on designing SCPGs for other gaits and different legged robots when target spikes are provided for the optimization process. Other criteria can be also added to the fitness functions based on SPIKE-distance, considering aspects of hardware implementation, for example.

## Author Contributions

AE has contributed on the conception and design of the work in the grammar design and software implementation phases, has acquired and analyzed the CPG’s topologies for hexapod locomotion, and has been involved on the draft process. HR-G has contributed on the conception and design of this work in the SNNs and locomotion gaits phases, has analyzed the resulting CPG topologies, and has worked on the draft process, contributing to a critical revision on the paper’s content and given his approval for the results. MC and MO-R have contributed on the conception of this work; they have been involved on the draft process, aiding with their critical revision and giving their final approval. EG-H has contributed on the conception and design of the work in the hexapod robot’ configuration and implementation of designed CPG over FPGAs phases, has analyzed the designed topologies over real hexapod robots, and has been involved on the draft process. MS-F has contributed on the conception and design of this work in the design part supporting the optimization phase and has been implicated from draft process, to the critical revisions for improving the content.

## Conflict of Interest Statement

The authors declare that the research was conducted in the absence of any commercial or financial relationships that could be construed as a potential conflict of interest.

## Figures and Tables

**Figure A1:**
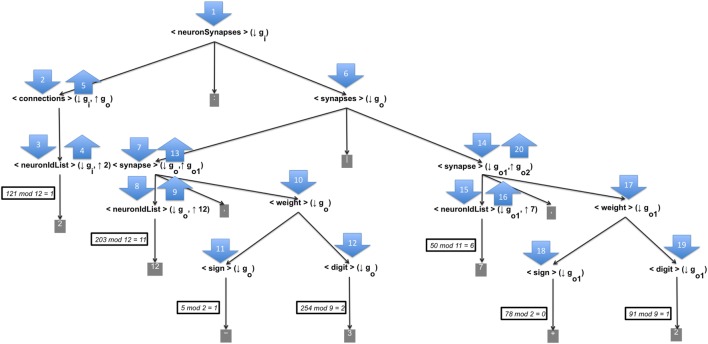
**Example of a derivation tree by the CGE’s mapping process**. For the input numeric string [121, 203, 5, 254, 50, 78, 91, 17, 31] the CGE’s mapping procees generates the word 2:12, −3|7, +2.

## Appendix

### A Connectivity Word Derivation Example

This appendix shows an example of the generation of a word that represents the presynaptic connections and their weight configuration through the CGE mapping process and the proposed grammar. The input numeric string is [121, 203, 5, 254, 50, 78, 91, 17, 31], Figure A1 shows the generated derivation tree for the input numeric string; the sequence of the derivation tree is marked by numerated up/down arrows.

According to the numeration of the arrows, the full process can be described as follows:

1. The axiom of the CGE, <*neuronSynapses*> (↓ *g_i_*) is processed. It only has one production, thus the axiom is replaced by its unique production and no codon is used. The status of the word until now is <*connections*> (↓ *g_i_*, ↑ *g_o_*): <*synapses*> (↓ *g_o_*).
2. The non-terminal <*connections*> (↓ *g_i_*, ↑ *g_o_*) is processed, due that is the leftmost non-terminal. As it has only one production, it is replaced by its production. The status of the word until now is <*neuronIdList*> (↓ *g_i_*, ↑ *n*): <*synapses*> (↓ *g_o_*).
3. Processing <*neuronIdList*> (↓ *g_i_*, ↑ *n*), it is necessary to choose one of the twelve productions. To select the production that replaces the current leftmost non-terminal, we consider the first number of the numeric string to applythe rule 121 *mod* 12 = 1; thus the second production is used to replace this non-terminal. The status of the word until now is 2: <*synapses*> (↓ *g_o_*).
4. The non-terminal <*neuronIdList*> (↓ *g_i_*, ↑ *n*) synthesizes a numeric value of 2 from the selected production which replaced it. This value will be used in an upper level of the derivation tree.
5. In this step, we apply the semantic rule of the non-terminal <*connections*> (↓ *g_i_*, ↑ *g_o_*). This rule carries out the synthesis of a new CG called *g_o_*, which contains all the information of the CG *g_i_* plus an unique production to the non-terminal <*synapses*> (↓ *g_o_*). The production contains the elements to generate the presynaptic configuration neuron. The number of elements is indicate by the synthesized number in previous steps.
6. The non-terminal <*synapses*> (↓ *g_o_*) is processed by using the synthesized CG *g_o_*, which already contains a unique production of this non-terminal. The status of the word until now is 2: <*synapse*> (↓ *g_o_*, ↑ *g_o_*_1_) | $<\mathrm{synapse}>\left(↓g_{o_{1}},↑g_{o_{2}}\right)$.
7. Processing $<\mathrm{synapse}>\left(↓g_{o},↑g_{o_{1}}\right)$, it is replaced by its unique production. The status of the word until now is 2: <*neuronIdList*> (↓ *g_o_*, ↑ *n*), <*weight*> (↓ *g_o_*) | $<\mathrm{synapse}>\left(↓g_{o_{1}},↑g_{o_{2}}\right)$.
8. The non-terminal <*neuronIdList*> (↓ *g_o_*, ↑ *n*) is processed by choosing one of its twelve productions. Applying the rule 203 *mod* 12 = 11, its last production is selected. The status of the word until now is 2:12, <*weight*> (↓ *g_o_*)|$<\mathrm{synapse}>\left(↓g_{o_{1}},↑g_{o_{2}}\right)$.
9. The non-terminal <*neuronIdList*> (↓ *g_o_*, ↑ *n*) synthesizes a numeric value of 11 from the selected production which replaced it. This value will be used in an upper level of the derivation tree.
10. The leftmost <*weight*> (↓ *g_o_*) is processed. It has only one production, thus it is replaced by its production. The status of the word until now is 2:12, <*sign*> (↓ *g_o_*) <*digit*> (↓ *g_o_*) | $<\mathrm{synapse}>\left(↓g_{o_{1}},↑g_{o_{2}}\right)$.
11. Processing <*sign*> (↓ *g_o_*) based on the rule 5*mod*2 = 1, its second production is selected to replace it. The status of the word until now is 2:12, − <*digit*> (↓ *g_o_*) | $<\mathrm{synapse}>\left(↓g_{o_{1}},↑\;g_{o_{2}}\right)$.
12. Processing <*digit*> (↓ *g_o_*) by applying the rule 254*mod*9 = 2, the third production is selected to replace it. The status of the word until now is 2:12, −3|$<\mathrm{synapse}>\left(↓g_{o_{1}},↑g_{o_{2}}\right)$.
13. In this step, it is applied the semantic rule of the non-terminal <*synapses*> (↓ *g_o_*). The rule synthesizes a new CG called $g_{o_{1}}$, which contains all the information of the CG *g_o_* less the production of <*neuronIdList*> (↓ *g_o_*, ↑ *n*) that synthesized the value of 12.
14. Processing $<\mathrm{synapse}>\left(↓g_{o_{1}},↑g_{o_{2}}\right)$ by using the recently sinthetized CG $g_{o_{1}}$, it is replaced by its unique production. The status of the word until now is 2:12, −3|$<\mathrm{neuronIdList}>\left(↓g_{o_{1}},↑n\right),<\mathrm{weight}>\left(↓g_{o_{1}}\right)$.
15. The non-terminal $<\mathrm{neuronIdList}>\left(↓g_{o_{1}},↑n\right)$ is processed by choosing one of its eleven productions. Applying the rule 50*mod*11 = 6, its seventh production is selected. The status of the word until now is 2:12, −3|7, $<\mathrm{weight}>\left(↓g_{o_{1}}\right)$.
16. The non-terminal $<\mathrm{neuronIdList}>\left(↓g_{o_{1}},↑n\right)$ synthesizes a numeric value of 7 from the selected production which replaced it. This value will be used in an upper level of the derivation tree.
17. The leftmost $<\mathrm{weight}>\left(↓g_{o_{1}}\right)$ is processed. It has only one production, thus it is replaced by its production. The status of the word until now is 2:12, −3|7, $<\mathrm{sign}>\left(↓g_{o_{1}}\right)<\mathrm{digit}>\left(↓g_{o_{1}}\right))$.
18. Processing $<\mathrm{sign}>\left(↓g_{o_{1}}\right)$ based on the rule 78*mod*2 = 0, its first production is selected to replace it. The status of the word until now is 2:12, −3|7, $+<\mathrm{digit}>\left(↓g_{o_{1}}\right))$.
19. Processing $<\mathrm{digit}>\left(↓g_{o_{1}}\right)$ by applying the rule 91*mod*9 = 1, the second production is selected to replace it. The status of the word until now is 2:12, −3|7, +2.
20. In this step, it is applied the semantic rule of the non-terminal $<\mathrm{synapses}>\left(↓g_{o_{1}}\right)$. The rule synthesized a new CG called $g_{o_{2}}$, which contains all the information of the CG $g_{o_{1}}$ less the production of <*neuronIdList*> (↓ *g_o_*, ↑ *n*) that synthesized the value of 7. But the derivation ends here and the rest of codons are not used.

### B Bivariate SPIKE-Distance

*The SPIKE-Distance* (Kreuz et al., 2013) is a parameter-free and timescale-adaptive measure for estimating the degree of synchrony between spike trains. In general, the distance is defined as a temporal average of the spike trains’ dissimilarity profiles. There are several variants of SPIKE-distance, for this study the Bivariate SPIKE-distance is used. The Bivariate SPIKE-distance is defined by equation (A1).

(A1) $$D_{S}\left(s_{1},s_{2}\right)=\frac{1}{T}\int_{t=0}^{T}S\left(t\right)\mathrm{dt}$$

where *T* is the overall length of the spike trains; for this study, it is 24 times (from time 0 to 23) for each spike train in all experiments. And *S*(*t*) is the dissimilarity profile between two spike trains. Before starting the dissimilarity profile calculations, the spike trains must be formatted as follows:

1. To add auxiliary leading spikes at time *t* = 0 and auxiliary trailing spikes at time *t* = *T* to each spike train; this is to avoid ambiguities in some calculations.
2. To label the spike times in the spike trains *n* = 1, 2 by $t_{i}^{n}$, *i* = 1, … , *M_n_*; the maximum number of spike times of the *n*th spike train is given by *M_n_*.

The computation of *S*(*t*) is based on four corner spikes surrounding the current time *t* (Mulansky et al., 2015), the preceding spikes $t_{P}^{\left(n\right)}\left(t\right)$ and the following spikes $t_{F}^{\left(n\right)}\left(t\right)$ where *n* = 1, 2; they are computedby using equations (A2) and (A3), respectively.

(A2) $$t_{P}^{\left(n\right)}\left(t\right)=\max\left(t_{i}^{\left(n\right)}|t_{i}^{\left(n\right)}\leq t\right)$$

(A3) $$t_{F}^{\left(n\right)}\left(t\right)=\min\left(t_{i}^{\left(n\right)}|t_{i}^{\left(n\right)}>t\right)$$

The inter-spike interval $x_{\mathrm{ISI}}^{\left(n\right)}\left(t\right)$, is given by equation (A4).

(A4) $$x_{\mathrm{ISI}}^{\left(n\right)}\left(t\right)=t_{F}^{\left(n\right)}\left(t\right)-t_{P}^{\left(n\right)}\left(t\right)$$

For each corner spikes, the distance to the closest spike of the other spike train is computed. Equations (A5) and (A6) show the distances for the spike train *n* = 1, this is similarly made with $\Delta t_{P}^{\left(2\right)}\left(t\right)$ and $\Delta t_{F}^{\left(2\right)}\left(t\right)$ for the spike train *n* = 2.

(A5) $$\Delta t_{P}^{\left(1\right)}\left(t\right)=\min\left(\left|t_{P}^{\left(1\right)}\left(t\right)-t_{i}^{\left(2\right)}\right|\right)$$

(A6) $$\Delta t_{F}^{\left(1\right)}\left(t\right)=\min\left(\left|t_{F}^{\left(1\right)}\left(t\right)-t_{i}^{\left(2\right)}\right|\right)$$

Next, the distance of the corner spikes to the current time is calculated by equations (A7) and (A8).

(A7) $$x_{P}^{\left(n\right)}\left(t\right)=t-t_{P}^{\left(n\right)}\left(t\right)$$

(A8) $$x_{F}^{\left(n\right)}\left(t\right)=t_{F}^{\left(n\right)}\left(t\right)-t$$

The weighted distance of each spike train *n* = 1, 2 is given by equation (A9).

(A9) $$S_{n}\left(t\right)=\frac{\Delta t_{P}^{\left(n\right)}\left(t\right)x_{F}^{\left(n\right)}\left(t\right)+\Delta t_{F}^{\left(n\right)}\left(t\right)x_{P}^{\left(n\right)}\left(t\right)}{x_{\mathrm{ISI}}^{\left(n\right)}\left(t\right)}$$

Finally, the disimilarity profile *S*(*t*) is calculated by equation (A10).

(A10) $$S\left(t\right)=\frac{S_{1}\left(t\right)x_{\mathrm{ISI}}^{\left(2\right)}\left(t\right)+S_{2}\left(t\right)x_{\mathrm{ISI}}^{\left(1\right)}\left(t\right)}{2{\left(x_{\mathrm{ISI}}^{\left(1\right)}\left(t\right)+x_{\mathrm{ISI}}^{\left(2\right)}\left(t\right)\right)}^{2}}$$

The Bivariate SPIKE-distance is not a metric as such, but it has useful features such as: it is bound to 0 ≤ *D_S_* ≤ 1, if *s*_1_ = *s*_2_ then *D_S_*(*s*_1_, *s*_2_) = 0 and the distance between *s*_1_ and *s*_2_ is symmetric *D_S_*(*s*_1_, *s*_2_) = *D_S_*(*s*_2_, *s*_1_).

### C Weight Matrixes

(A11) $$W_{w}=\left|\begin{array}{cccccccccccc} 0 & 4 & 0 & 0 & 0 & 0 & 0 & 0 & 0 & 0 & 0 & 0\\ 0 & 0 & 0 & 0 & 0 & 0 & 0 & 0 & 0 & 0 & 9 & 0\\ 0 & 0 & 0 & 3 & 0 & 0 & 0 & 0 & 0 & 0 & 0 & 0\\ 0 & 0 & 0 & 0 & 1 & 0 & 0 & 0 & 0 & 0 & 0 & 0\\ 0 & 0 & 0 & 0 & 0 & 0 & 0 & 7 & 0 & 0 & 0 & 0\\ 0 & 0 & 0 & 0 & 0 & 0 & 0 & 0 & 6 & 0 & 0 & 0\\ 0 & 0 & 0 & 0 & 0 & 1 & 0 & 0 & 0 & 0 & 0 & 0\\ 7 & 0 & 0 & 0 & 0 & 0 & 0 & 0 & 0 & 0 & 0 & 0\\ 0 & 6 & 0 & 0 & 0 & 0 & 0 & 0 & 0 & 0 & 0 & 0\\ 0 & 0 & 3 & 0 & 0 & 0 & 0 & 0 & 0 & 0 & 0 & 0\\ 0 & 0 & 0 & 6 & 0 & 0 & 0 & 0 & 0 & 0 & 0 & 0\\ 0 & 0 & 0 & 0 & 0 & 0 & 5 & 0 & 0 & 0 & 0 & 0 \end{array}\right|$$

(A12) $$W_{j}=\left|\begin{array}{cccccccccccc} 0 & 5 & 0 & 0 & 0 & 0 & 0 & 0 & 0 & 0 & 0 & 0\\ 0 & 0 & 5 & 0 & 0 & 0 & 0 & 0 & 0 & 0 & 0 & 0\\ 0 & 0 & 0 & 0 & 0 & 0 & 0 & 1 & 0 & 0 & 0 & 0\\ 0 & 0 & 0 & 0 & 0 & 0 & 0 & 0 & 7 & 0 & 0 & 0\\ 0 & 0 & 0 & 0 & 0 & 0 & 0 & 0 & 0 & 9 & 0 & 0\\ 0 & 0 & 0 & 0 & 0 & 0 & 0 & 0 & 0 & 0 & 8 & 0\\ 0 & 0 & 0 & 2 & 0 & 0 & 0 & 0 & 0 & 0 & 0 & 0\\ 0 & 0 & 0 & 0 & 5 & 0 & 0 & 0 & 0 & 0 & 0 & 0\\ 0 & 0 & 0 & 0 & 0 & 9 & 0 & 0 & 0 & 0 & 0 & 0\\ 8 & 0 & 0 & 0 & 0 & 0 & 0 & 0 & 0 & 0 & 0 & 0\\ 0 & 2 & 0 & 0 & 0 & 0 & 0 & 0 & 0 & 0 & 0 & 0\\ 0 & 0 & 0 & 0 & 0 & 0 & 4 & 0 & 0 & 0 & 0 & 0 \end{array}\right|$$

(A13) $$W_{r}=\left|\begin{array}{cccccccccccc} 0 & 0 & 0 & 0 & 0 & 1 & 0 & 0 & 0 & 0 & 0 & 0\\ 0 & 0 & 0 & 0 & 0 & 0 & 9 & 0 & 0 & 0 & 0 & 0\\ 0 & 0 & 0 & 0 & 0 & 0 & 0 & 0 & 0 & 0 & 0 & 7\\ 0 & 0 & 0 & 0 & 0 & 0 & 0 & 0 & 4 & 0 & 0 & 0\\ 0 & 0 & 0 & 0 & 0 & 5 & 0 & 0 & 0 & 0 & 0 & 0\\ 0 & 0 & 0 & 0 & 0 & 0 & 6 & 0 & 0 & 0 & 0 & 0\\ 0 & 0 & 0 & 4 & 0 & 0 & 0 & 0 & 0 & 0 & 0 & 0\\ 0 & 0 & 0 & 0 & 0 & 0 & 0 & 0 & 5 & 0 & 0 & 0\\ 0 & 0 & 0 & 0 & 0 & 1 & 0 & 0 & 0 & 0 & 0 & 0\\ 0 & 0 & 5 & 0 & 0 & 0 & 0 & 0 & 0 & 0 & 0 & 0\\ 0 & 0 & 0 & 0 & 0 & 0 & 0 & 9 & 0 & 0 & 0 & 0\\ 9 & 0 & 0 & 0 & 0 & 0 & 0 & 0 & 0 & 0 & 0 & 0 \end{array}\right|$$

(A14) $$\mathrm{Waio}=\left|\begin{array}{cccccccccccc} 0 & 8 & 0 & 0 & 0 & 0 & 0 & 0 & 0 & 0 & 0 & 0\\ -7 & 0 & 4 & 2 & -2 & 0 & 1 & 2 & 0 & 0 & 2 & 0\\ -9 & -5 & 0 & 8 & 4 & 1 & 0 & -2 & 4 & 0 & 0 & 0\\ 0 & 0 & 0 & 0 & 8 & 0 & 0 & 0 & 0 & 0 & 0 & 0\\ 0 & 0 & 0 & 0 & 0 & 5 & 0 & 0 & 0 & 0 & 0 & 0\\ 6 & 0 & 0 & 0 & -9 & 0 & 0 & 0 & 3 & 0 & 3 & -1\\ 0 & 0 & 0 & 0 & 0 & 0 & 0 & 5 & 0 & 0 & 0 & 0\\ 0 & 0 & 0 & 0 & 0 & 0 & 0 & 0 & 4 & 0 & 0 & 0\\ 0 & 0 & 0 & 0 & 0 & 0 & 0 & 0 & 0 & 8 & 0 & 0\\ 0 & 0 & 0 & 0 & 0 & 0 & 0 & 0 & 0 & 0 & 9 & 0\\ 0 & -4 & 0 & 0 & 0 & -3 & 0 & -2 & 0 & 0 & 3 & 9\\ 0 & 1 & 0 & -3 & 8 & -1 & 5 & 0 & -4 & 0 & -4 & 0 \end{array}\right|$$
